# Liver venous deprivation prior to hepatectomy: an interventional radiology procedure

**DOI:** 10.1590/0100-3984.2019.0124

**Published:** 2021

**Authors:** Vinicius de Pádua Vieira Alves, André Azevedo, Danilo Alves de Araujo, Leonardo Azevedo Marcondes Rodrigues, Feliciano Silva de Azevedo

**Affiliations:** 1 Universidade Federal Fluminense (UFF), Niterói, RJ, Brazil.; 2 Department of Interventional Radiology, Americas Medical City, Rio de Janeiro, RJ, Brazil.

## INTRODUCTION

In many cases, hepatectomy is the only option for long-term survival in patients with primary or secondary liver malignancies. When a tumor proves unresectable because the resection will result in insufficient remnant liver volume, portal vein embolization (PVE) can be performed to stimulate future liver growth, the objective being to avoid post-hepatectomy liver failure^(1)^. Although PVE is safe and effective, it does not always induce sufficient hypertrophy of the future remnant liver, even after a significant time. Consequently, several other approaches have been proposed as alternatives to PVE^(2)^. The most recently developed of such approaches is liver venous deprivation (LVD), a technique that combines hepatic vein embolization of the right hepatic vein (RHV) and accessory RHV, if present and PVE. Among the various interventional procedures that could be used for this purpose, LVD has been shown to promote the fastest and greatest increase in the future remnant liver volume, the reported growth at 14 days being 62.5% after LVD, compared with 8-27% after PVE^(2-4)^.

Here, we describe the case of a 70-year-old patient diagnosed with Bismuth type IIIa cholangiocarcinoma. The patient was treated successfully with LVD prior to hepatectomy.

## LVD TECHNIQUE

The patient underwent computed tomography (CT) with angiography and volumetric analysis of the liver, processed with specific software (IntelliSpace Portal 9.0; Philips Health Care, Eindhoven, The Netherlands), 30 days before the procedure in order to determine the remnant liver volume, which was found to be insufficient. Hepatectomy was also contraindicated because the patient showed an elevated bilirubin level, and an internal-external biliary catheter was therefore inserted for. The patient received antibiotic prophylaxis with a single dose (3 g) of ampicillin/sulbactam. Because of the long duration of the procedure and need for cooperation, as well as the possible benefit of apnea to facilitate punctures, general anesthesia was administered.

**Figure 1.**
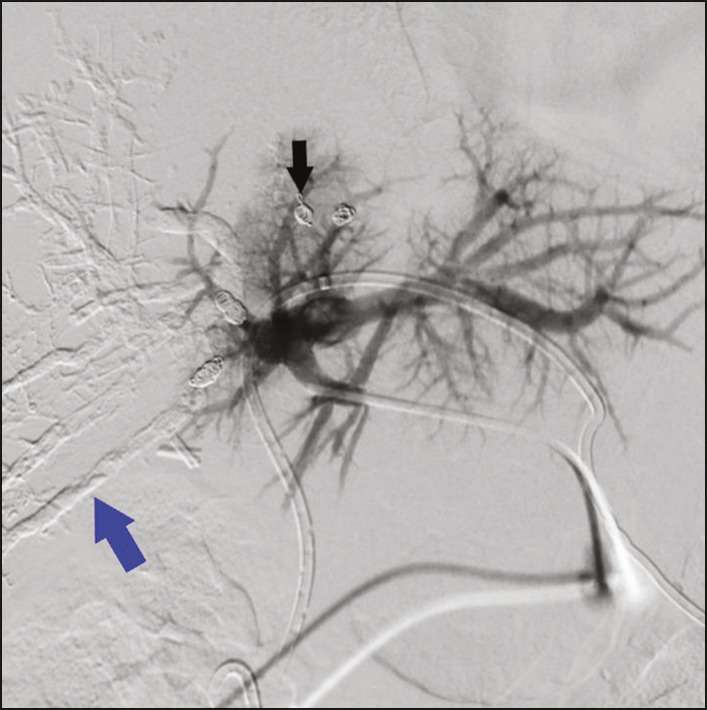
Direct portography after RPV embolization, showing where the RPV was embolized with Histoacryl (blue arrow) and coils (black arrow). Note the interrupted portal flow to the right lobe.

The intervention begins with embolization of the right portal vein (RPV). A decision must be made as to whether access should be gained through the RPV, with the disadvantage of requiring a countercurrent catheter to deliver the embolization materials, or through the left portal vein (LPV), our preferred method, which, despite the manipulation of the future remnant liver, grants direct access to the RPV. The LPV was accessed under ultrasound guidance with a percutaneous access set, containing a 15-cm 22-gauge Chiba needle, a 0.018-in. nitinol guidewire, and a 0.038-in. introducer. The distal part of the LPV was punctured with the Chiba needle and opacified. The nitinol guidewire was then introduced through the needle to gain initial access. The Chiba needle was then removed, and the access set introducer was advanced over the wire, after which a stiff 0.035-in. hydrophilic guidewire was advanced over the introducer. A 5 Fr angiographic catheter was then advanced over the introducer and the stiff guidewire into the portal trunk, where portography was performed. We then performed selective catheterization of the segmental RPV and posterior embolization of the portal branches of segments V, VI, VII, and VIII, one by one with n-butyl-2-cyanoacrylate (Glubran 2; GEM SRL, Viareggio, Italy) and iodized oil (Lipiodol; Guerbet, Villepinte, France), at a ratio of 1:2 or 1:3 when injected via a 4 Fr or 5 Fr catheter and 1:4 or 1:5 when injected via a microcatheter. That was followed by selective microcatheterization of the portal branches of segment IVa, distal embolization with microspheres, and proximal embolization with a metal coil (Figure 1).

**Figure 2:**
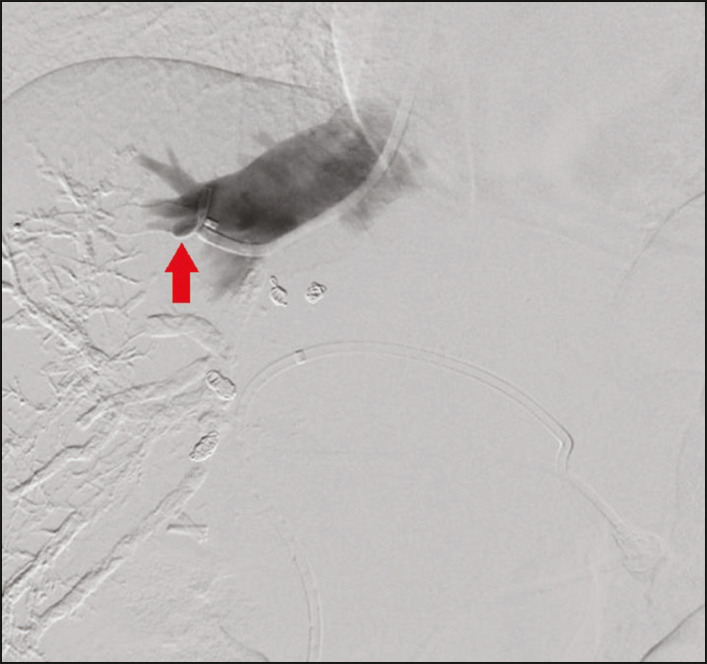
Hepatic venography showing the vascular plug (arrow) in the RHV and the interrupted venous flow in the vessel.

**Figure 3:**
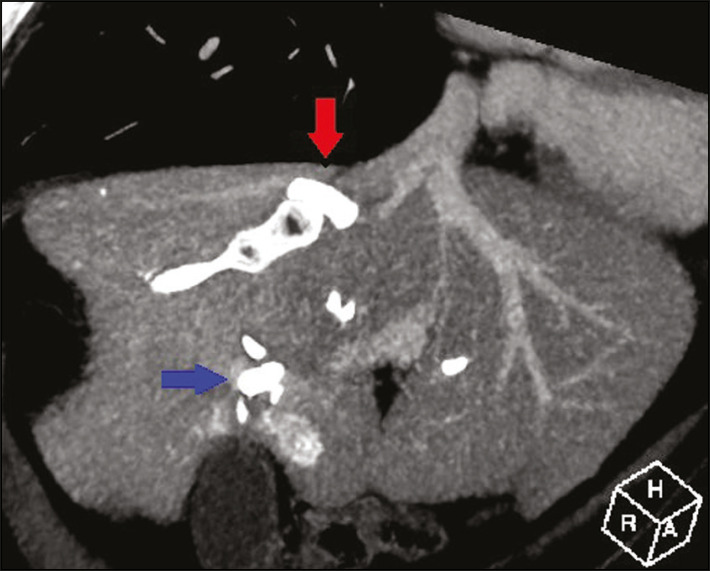
Reconstruction of contrast-enhanced oblique coronal venous phase CT scan, acquired after LVD, showing the RHV embolized with the vascular plug (red arrow) and some portal branches embolized with Histoacryl and Lipiodol (blue arrow).

**Figure 4:**
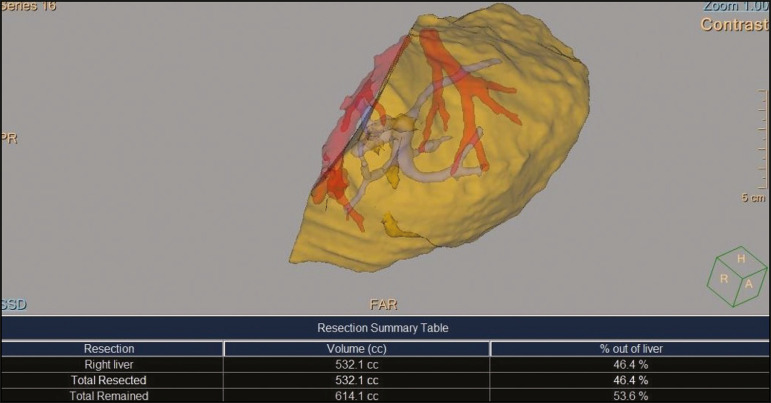
Volumetric analysis of the liver, performed 30 days after LVD, demonstrating that the total liver volume was 1146 cc and that 532.1 cc (46.4%) of that volume was embolized.

After RPV embolization, the hepatic vein embolization was initiated. The hepatic veins can be accessed via a transparietal approach or a transjugular approach. At our institution, the transjugular approach is preferred. An ultrasound-guided right internal jugular vein puncture was performed with a micropuncture access set kit. After the introducer was in place, a 5 Fr diagnostic catheter and hydrophilic 0.035-in. guidewire were passed through, RHV was catheterized, and pre-embolization venography was performed. A stiff Teflon guidewire was then introduced, and 7 Fr Multiport guide catheter was introduced up to the RHV. Through that guide catheter, an 18 mm × 22 mm vascular plug was deployed with its distal part 10 mm before the junction with the lower superior vena cava, to facilitate subsequent surgical ligature of the hepatic vein, and the RHV was embolized (Figures 2 and 3). It is of note that the plug chosen is oversized by at least 50%, which substantially reduces the risk of migration^(2)^. A control CT with volumetric analysis of the liver (Figures 3 and 4) was performed 31 days after the intervention, and the patient underwent hepatectomy 10 days after the control CT. Therefore, the total time from embolization to surgery was 41 days.
